# Experimental Implementation of NSER Mobile App for Efficient Real-Time Sharing of Prehospital Patient Information With Emergency Departments: Interrupted Time-Series Analysis

**DOI:** 10.2196/37301

**Published:** 2022-07-06

**Authors:** Kiyomitsu Fukaguchi, Tadahiro Goto, Tadatsugu Yamamoto, Hiroshi Yamagami

**Affiliations:** 1 Department of Emergency Medicine Shonan Kamakura General Hospital Kamakura-shi, Kanagawa Japan; 2 TXP Research TXP Medical Co Ltd Tokyo Japan

**Keywords:** emergency department, emergency medical services, mobile apps, interrupted time series analysis, emergency, patient record, implementation, patient care, app, implement, medical informatics, clinical informatics, decision support, electronic health record, eHealth, digital health

## Abstract

**Background:**

With the aging society, the number of emergency transportations has been growing. Although it is important that a patient be immediately transported to an appropriate hospital for proper management, accurate diagnosis in the prehospital setting is challenging. However, at present, patient information is mainly communicated by telephone, which has a potential risk of communication errors such as mishearing. Sharing correct and detailed prehospital information with emergency departments (EDs) should facilitate optimal patient care and resource use. Therefore, the implementation of an app that provides on-site, real-time information to emergency physicians could be useful for early preparation, intervention, and effective use of medical and human resources.

**Objective:**

In this paper, we aimed to examine whether the implementation of a mobile app for emergency medical service (EMS) would improve patient outcomes and reduce transportation time as well as communication time by phone (ie, phone-communication time).

**Methods:**

We performed an interrupted time-series analysis (ITSA) on the data from a tertiary care hospital in Japan from July 2021 to October 2021 (8 weeks before and 8 weeks after the implementation period). We included all patients transported by EMS. Using the mobile app, EMS can send information on patient demographics, vital signs, medications, and photos of the scene to the ED. The outcome measure was inpatient mortality and transportation time, as well as phone-communication time, which was the time for EMS to negotiate with ED staffs for transport requests.

**Results:**

During the study period, 1966 emergency transportations were made (n=1033, 53% patients during the preimplementation period and n=933, 47% patients after the implementation period). The ITSA did not reveal a significant decrease in patient mortality and transportation time before and after the implementation. However, the ITSA revealed a significant decrease in mean phone-communication time between pre- and postimplementation periods (from 216 to 171 seconds; −45 seconds; 95% CI −71 to −18 seconds). From the pre- to postimplementation period, the mean transportation time from EMS request to ED arrival decreased by 0.29 minutes (from 36.1 minutes to 35.9 minutes; 95% CI −2.20 to 1.60 minutes), without change in time trends. We also introduced cases where the app allowed EMS to share accurate and detailed prehospital information with the emergency department, resulting in timely intervention and reducing the burden on the ED.

**Conclusions:**

The implementation of a mobile app for EMS was associated with reduced phone-communication time by 45 seconds (22%) without increasing mortality or overall transportation time despite the implementation of new methods in the real clinical setting. In addition, real-time patient information sharing, such as the transfer of monitor images and photos of the accident site, could facilitate optimal patient care and resource use.

## Introduction

With the aging society, the number of emergency transportation has been growing [1]. Researchers used a nationwide database in Japan and reported that the annual emergency transportations increased from 4 million in 2000 to 6 million in 2020 [1]. The substantial increase in emergency transportation causes depletion of medical resources and emergency department (ED) overcrowding, resulting in poor patient outcomes [2,3]. Indeed, in Japan, approximately 10,000 emergency patients were turned away by at least 6 hospitals annually [4].

When a patient has stroke, acute myocardial infarction, or severe trauma, it is important to transport them to an appropriate hospital in order to provide appropriate management immediately [5-9]. Although accurate diagnosis in the prehospital setting is challenging, real-time sharing of detailed patient information should facilitate appropriate transportation and management [10,11]. However, at present, patient information is mainly communicated by telephone, which has the potential risk of communication errors such as mishearing. In addition, photos of the patient, patient monitors (eg, electrocardiogram), and the accident scene can contain important information that emergency physicians would want to know in advance. Therefore, the implementation of an app that provides on-site, real-time information to emergency physicians could be useful for early preparation, intervention, and effective use of medical and human resources [5-8,10-12].

Thus, in this study, we examined whether the implementation of a mobile app for emergency medical services (EMS) could improve patient outcomes. We hypothesized that patient mortality would be improved by shortening the transportation time and time for EMS to negotiate with ED staffs for transport requests.

## Methods

### Study Design and Settings

We performed an interrupted time-series analysis to examine the change in outcomes before and after the introduction of a mobile app for EMS. This study was conducted using data on EMS transportations by Kamakura City Fire Department to Shonan Kamakura General Hospital, a tertiary care hospital in Japan, from July 8, 2021, to October 27, 2021. Kamakura City is a city in Kanagawa Prefecture, Japan, with a population of 172,948 people and an average age of 48.8 years as of 2020. The city has a high ratio of the aging population, with 53,517 (31.1%) people aged ≥65 years. Kamakura City has 8 fire departments that make 29.9 emergency transportations per day and 10,896 per year [13]. The Emergency Department of Shonan Kamakura General Hospital had 43,506 ED visits by patients in 2020. Among these patients, 14,925 (34.3%) were transported to the emergency department by EMS, which was the highest annual number of emergency transportations of all hospitals in Japan that year. All patients transported by EMS were accepted [14]. The number of patients transported by the Kamakura City EMS during the study period (16 weeks) was estimated to be approximately 2000 based on the number of patients transported in the past.

### Ethics Approval

This study protocol was approved by the Ethics Committee of Shonan Kamakura General Hospital (approval number: TGE01663-024; approval date: February 25, 2021). The need for informed consent was waived due to the retrospective nature of the study. For the publication of images and personal information, the patients’ consent was obtained, and their consent was recorded in their medical charts. The institutional review board approved the use of patient information for this study.

### Apps Used and Their Features

We implemented an app for EMS named NEXT Stage ER mobile (NSER mobile) on September 2, 2021. The NSER mobile is an app that was developed to reduce the complexity of information entry for EMS teams and to increase the efficiency of information sharing with EDs. The app is equipped with high-precision voice input and optical character recognition functions that enable timely information sharing by sending information to hospitals in the original text (eg, medications and past medical history) and in coded form (eg, International Statistical Classification of Diseases and Related Health Problems, 10th revision codes). We provide a description of the usage process of the NSER mobile by EMS and hospitals in Multimedia Appendices 1 and 2. All EMS staffs took 3-hour lectures to learn to use the app before implementation.

The developer of NSER mobile is TXP Medical Co Ltd. A free trial for this product is available through the website of TXP Medical [15]. The system’s accuracy and validity are now being examined in a multicenter study (UMIN-CTR ID: UMIN000045775), but it is fundamentally the same as the NEXT Stage ER system, which is a well-validated ED information system [16]. In addition, in this study, we did not use table data from the app. All data are abstracted from the Kamakura City Fire Department and patient registry of ED patients of Shonan Kamakura General Hospital. Thus, the only data we extracted from the NEXT Stage ER mobile system for this study are images (though more detailed data exist in the cloud server of the system).

### Study Population

All patients transported to Shonan Kamakura General Hospital by the Kamakura City EMS during the study period were included in the study. Exclusion criteria were cases with missing data on call times between the hospital and EMS and EMS activity times (26/1992, 1.3%).

### Outcome Measures

The primary outcome was inpatient mortality. Secondary outcomes were the overall transportation time (the time from the patient’s call for an ambulance to arrival at the hospital), and phone-communication time (the time for EMS to negotiate with ED staffs for transport requests).

### Statistical Analysis

Cases with missing data on the time spent in EMS and the emergency department (ED) were excluded from the analysis (26/1992, 1.3%). Using data that met the inclusion criteria, we performed an interrupted time-series analysis with a linear regression model to examine whether the implementation of the mobile app had an impact on the outcomes. An interrupted time-series analysis is a quasi-experimental design for evaluating the effectiveness of population-level health interventions implemented at a clearly defined point in time and is thus widely used to evaluate the effectiveness of interventions [17-19]. The time unit was weeks (ie, 8 time points before and 8 time points after implementation).

Additionally, we described specific cases where the immediate sharing of patient information and photos with NSER mobile led to rapid diagnosis and intervention.

All statistical analyses were conducted using R version 4.1.1 (R Foundation for Statistical Computing).

## Results

Among the 1992 patients transported by EMS, we excluded 26 patients (1.3%) with missing data on the transport time or the phone communication time, and the remaining 1966 (98.7%) patients were eligible for this study. The mean age was 66.7 years (SD 25.4), and 49% (n=963) were men. Of the 1966 patients, 1033 (53%) were transported during the preimplementation period, and 933 (47%) were transported in the postimplementation period. The patient characteristics in the pre- and postimplementation periods were similar. Additionally, there were no significant differences in the region of transportation and the EMS team numbers (Table 1).

**Table 1:** Characteristics of patients transported by emergency medical services.

Patient characteristics and variables		Patients transported using the app (n=1033)	Patients transported in the usual way (n=933)		*P* value	
Age (years), mean (SD)		65.8 (27.2)	68.1 (23.5)		.13	
**Age profile (years), n (%)**						
	≤18	98 (9.5)	48 (5.1)			
	18-64	307 (29.7)	232 (24.9)			
	65-84	324 (31.4)	343 (36.8)			
	≥85	304 (29.4)	310 (33.2)			
**Sex, n (%)**					.80	
	Male	508 (49.2)	464 (49.7)			
	Female	525 (50.8)	469 (50.3)			
Number of calls to the hospital, mean (SD)		1.07 (0.28)	1.05 (0.25)		.05	
**Region of emergency medical services, n (%)**						.17
	Kamakura	162 (15.7)	147 (15.8)			
	Ofuna	176 (17.0)	181 (19.4)			
	Fukasawa	203 (19.7)	155 (16.6)			
	Tamanawa	139 (13.4)	128 (13.7)			
	Koshigoe	121 (11.7)	109 (11.7)			
	Imaizumi	74 (7.2)	82 (8.8)			
	Shitirigahama	91 (8.8)	60 (6.4)			
	Zyoumyouzi	67 (6.5)	71 (7.6)			
**Level of consciousness (JCS^a^), n (%)**						.70
	0	524 (50.7)	443 (47.5)			
	1	163 (15.8)	178 (19.1)			
	2	143 (13.8)	123 (13.2)			
	3	90 (8.7)	79 (8.5)			
	10	47 (4.5)	45 (4.8)			
	20	14 (1.4)	15 (1.6)			
	30	5 (0.5)	4 (0.4)			
	100	7 (0.7)	8 (0.9)			
	200	7 (0.7)	4 (0.4)			
	300	33 (2.9)	34 (3.6)			
**Vital signs, mean (SD)**						
	Systolic blood pressure (mmHg)	133 (45.9)	135 (50.6)		.35	
	Diastolic blood pressure (mmHg)	79.4 (27.8)	79.6 (30.7)		.91	
	Pulse rate (per min)	89.1 (29.3)	84.5 (27.2)		<.001	
	Respiratory rate (per min)	21.2 (6.33)	20.8 (5.67)		.08	
	Saturation (%)	89.2 (24.5)	87.9 (26.8)		.25	
	Body temperature (°C)	35.0 (8.43)	34.4 (9.04)		.16	
**Classification of diseases^b^, n (%)**						<.001
	Endogenous disease	748 (72.4)	680 (72.9)			
	Trauma	270 (26.1)	234 (25.1)			
	Cardiac arrest	15 (1.5)	19 (2.0)			
**Severity of illness at the ED^c^ visit^b^, n (%)**						.70
	Minor illness	375 (36.3)	351 (37.6)			
	Moderate illness	567 (54.9)	492 (52.7)			
	Serious illness	76 (7.3)	71 (7.6)			
	Death	15 (1.5)	19 (2.0)			
**Disposition at the ED, n (%)**						.04
	Discharge	657 (63.6)	549 (58.8)			
	Admission	308 (29.8)	295 (31.6)			
	Transfer to another hospital for admission	50 (4.8)	68 (7.3)			
	Death	18 (1.7)	21 (2.3)			
**Prognosis, n (%)**						.56
	In-hospital death	53 (5.1)	46 (4.9)			

^a^JCS: Japan Coma Scale.

^b^The severity of illness at the emergency department is classified as follows: minor illness—patient can return home after treatment; moderate illness—patient requires inpatient treatment, but the disease severity is low and can be managed in a general ward; serious illness—multiorgan failures such as respiratory or circulatory failure requiring monitoring, a ventilator, vasopressors such as catecholamines, and admission to an intensive care unit; death—cardiac arrest on arrival at the hospital.

^c^ED: emergency department.

### Inpatient Mortality

**Figure 1:**
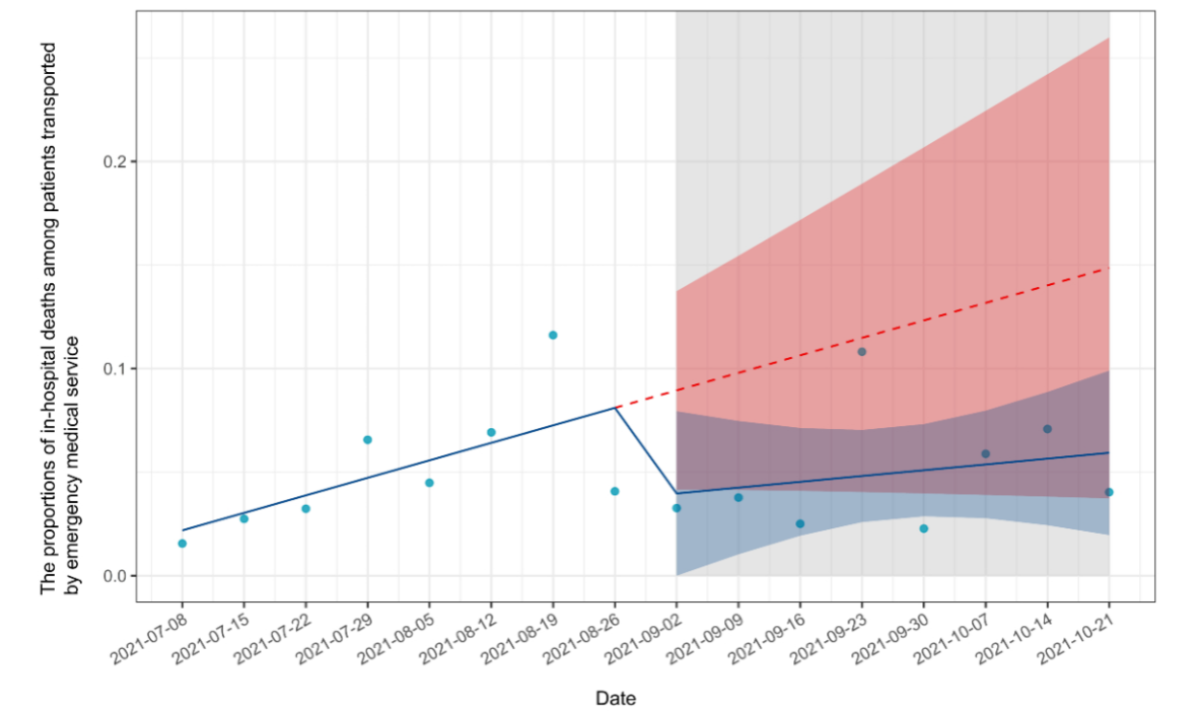
Interrupted time-series analysis of inpatient mortality.

Of the 1966 eligible patients, 53 (5.1%) died in hospital in the preimplementation period and 46 (4.9%) died in hospital in the postimplementation period. From the pre- to postimplementation period, the proportions of in-hospital deaths among patients who were transported to EDs during each period decreased by 5% (95% CI −11% to 1%), followed by a decreasing trend relative to preimplementation of −1% per week (95% CI −2% to 1%). There was no significant change in inpatient mortality. Figure 1 shows the proportions of in-hospital deaths among patients who were transported to emergency departments during each period. On September 2, 2021, EMS began transportation using the new app in place of the traditional method. The proportion of in-hospital deaths among the transported patients is plotted for 8-week periods before and after the implementation. For in-hospital mortality, the *R*^2^ for the preimplementation model was 0.26, while the *R*^2^ for the postimplementation model was 0.23. The results of interrupted time series analysis on inpatient mortality are shown in Table 2.

**Table 2:** Results of interrupted time-series analysis on inpatient mortality.

Time-series analysis	Estimate (95% CI)	*P* value
Trends in inpatient mortality before implementation	0.01 (0.00 to 0.02)	.07
Absolute change in the inpatient mortality before and after implementation	−0.05 (−0.11 to 0.01)	.11
Trends in inpatient mortality after implementation	0.00 (−0.01 to 0.01)	.50
Change in slope before and after implementation	−0.01 (−0.02 to 0.01)	.40

### The Transportation Time From EMS Request to ED Arrival

**Figure 2:**
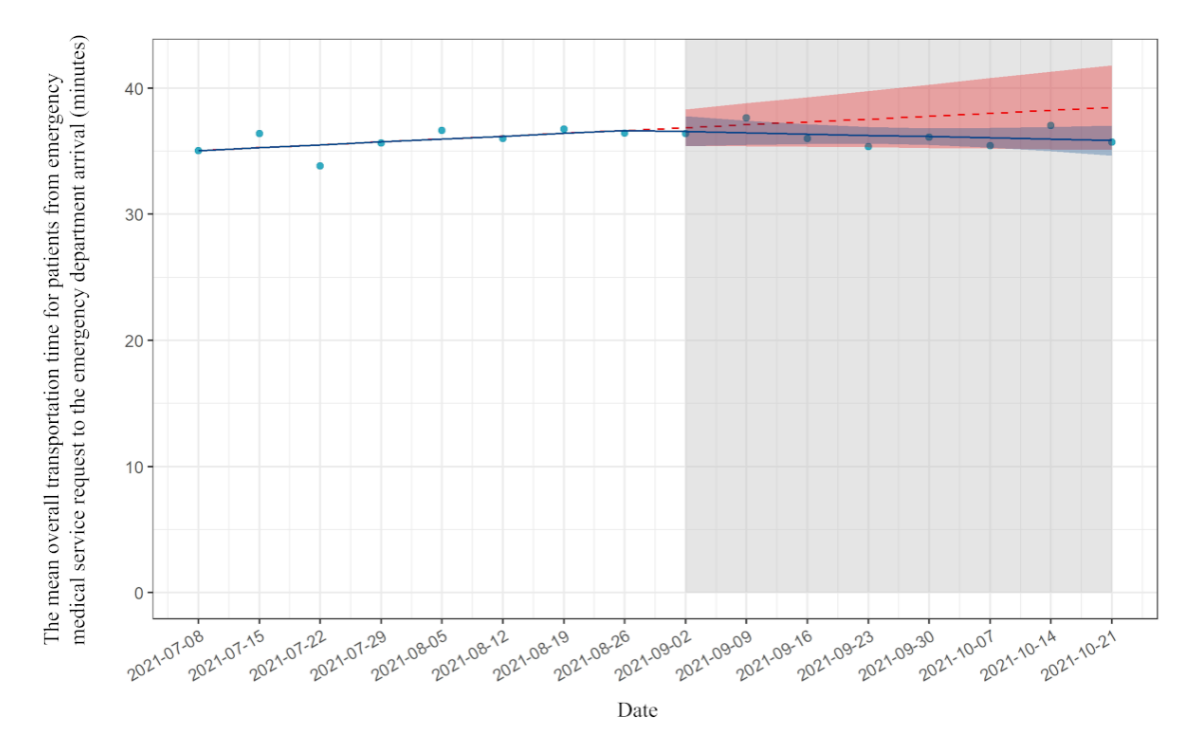
Interrupted time-series analysis on transportation time from emergency medical services to emergency department arrival.

The mean transportation time from EMS request to ED arrival was 35.9 minutes (SD 9.7 minutes) in the preimplementation period and 36.1 minutes (SD 8.5 minutes) in the postimplementation period. From the pre- to postimplementation period, the mean transportation time from EMS request to ED arrival decreased by 0.29 minutes (95% CI −2.20 to 1.60 minutes), followed by a decreasing trend relative to preimplementation of −0.33 minutes per week (95% CI −0.74 to 0.07; Table 3). Figure 2 shows the mean transportation time from the emergency call to arrival at the hospital. On September 2, 2021, EMS began transporting using the new app in place of the traditional method. The mean transportation time is plotted for 8-week periods before and after the implementation. For transportation time, the *R*^2^ for the preimplementation model was 0.30, and the *R*^2^ for the postimplementation model was 0.28.

**Table 3:** Results of interrupted time-series analysis on transportation time from emergency medical services to emergency department arrival.

Time-series analysis	Estimate (min), 95% CI	*P* value
Trends in mean transportation time before implementation	0.23 (−0.06 to 0.51)	.11
Absolute change in the transportation time before and after implementation	−0.29 (−2.20 to 1.60)	.70
Trends in mean transportation time after implementation	−0.10 (−0.39 to 0.18)	.40
Change in slope before and after implementation	−0.33 (−0.74 to 0.07)	.10

### Phone-Communication Time Between EMS Teams and Hospital

**Figure 3:**
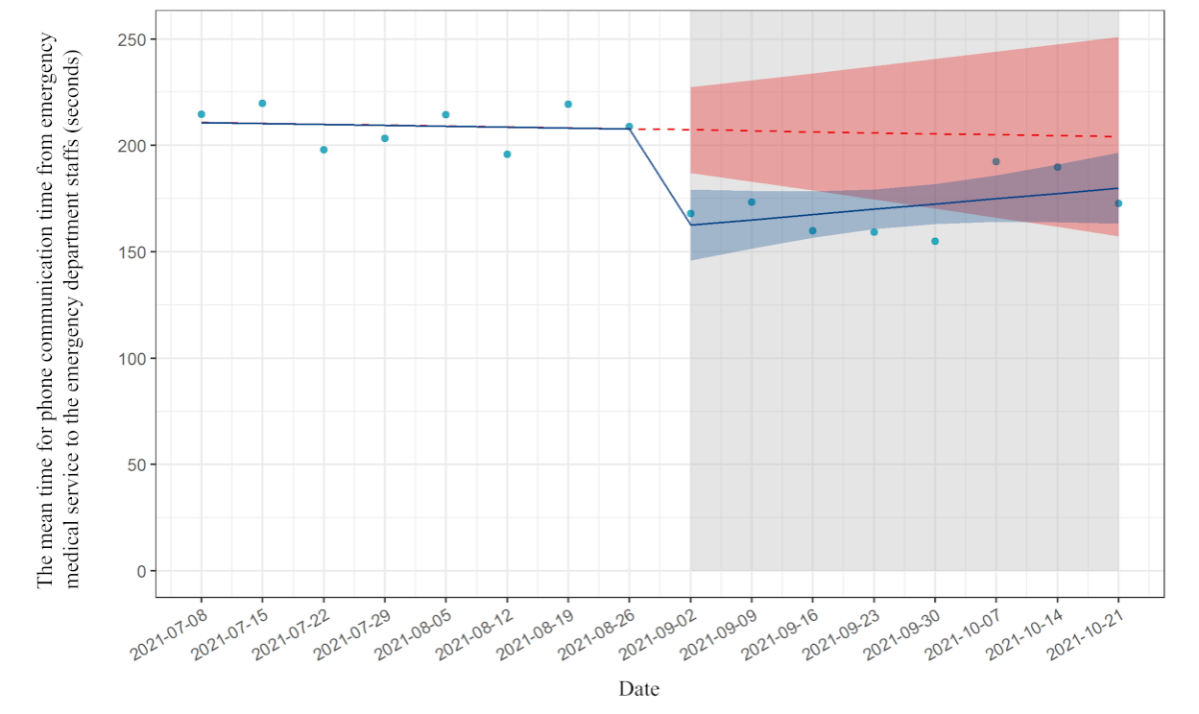
Interrupted time-series analysis on phone-communication time.

The mean time of phone communication between EMS and ED staffs was 216 (SD 107) seconds in the preimplementation period and 171 (SD 120) seconds in the postimplementation period. From the pre- to postimplementation period, the phone-communication time decreased by 45 seconds (95% CI −71 to −18 seconds), followed by an increasing trend relative to preimplementation of +2.9 seconds per week (95% CI −2.7 to 8.6; Table 4). The mean phone-communication time between EMS and the hospital is shown in Figure 3. On September 2, 2021, the EMS started using the app for transportation in place of the conventional method. The mean phone-call time is plotted for 8-week periods before and after the implementation. For phone-communication time, the *R*^2^ for the preimplementation model was 0.78, while the *R*^2^ for the postimplementation model was 0.72.

**Table 4:** Results of interrupted time-series analysis on phone-communication time.

Time-series analysis	Estimate (s), 95% CI	*P* value
Trends in mean phone-communication time before implementation	−0.44 (−4.4 to 3.6)	.80
Absolute change in the phone-communication time before and after implementation	−45.0 (−71.0 to −18.4)	.003
Trends in mean phone-communication time after implementation	2.5 (−1.5 to 6.5)	.20
Change in slope before and after implementation	2.9 (−2.7 to 8.6)	.30

### Specific Cases

We experienced several cases where the immediate sharing of patient information and photos using the NSER mobile has led to rapid diagnosis and intervention. For example, a 47-year-old man with a history of diabetes mellitus, who had visited the hospital the day before for chest pain, called for EMS again complaining of persistent chest pain. Upon EMS’s arrival, his electrocardiography monitor showed ST-segment elevation in lead Ⅱ (Multimedia Appendix 3). Before arriving at the hospital, he was diagnosed with ST-segment elevation acute myocardial infarction. Upon consulting with the cardiologist, he was able to start percutaneous coronary intervention within ten minutes after the patient’s arrival at the ED. Multimedia Appendix 3 shows the monitor screen in the ambulance transporting a 47-year-old man who called EMS complaining of chest pain. Lead Ⅱ showed ST-segment elevation, and the images were transmitted from EMS to the ED staff, leading to early diagnosis of acute myocardial infarction and percutaneous coronary intervention within 10 minutes of arrival at the hospital.

In another example, a 35-year-old man was riding a bicycle when he collided with a car traveling at 60 kilometers per hour. He had no memory when he was injured. While the mechanism of injury suggests a highly serious one, real-time information sharing (initial assessment of the patient, vital signs, photos of the injury scene, and damage to the bicycle) allowed us to determine that the injury was minor, with only a contusion on the left lower leg and the scalp. Consequently, we were able to reduce unnecessary preparation for the initial treatment, including surgery for damage control (Multimedia Appendices 4-6).

## Discussion

### Principal Findings

In this ITSA study, there was no substantial decrease in in-hospital mortality and overall transportation time between pre- and postimplementation periods. On the other hand, the implementation reduced phone-communication time by 22% without increasing mortality or overall transportation time despite the implementation of new methods in the real clinical setting.

The conventional communication between EMS and ED staff by telephone only poses substantial stress on EMS staff, exemplified by difficulty hearing and misidentification of information. Data related to patient demographics, vital signs, past medical history, and medications are likely misclassified due to such technical difficulties, and real-time information sharing could reduce such miscommunication. In addition, the app’s feature to share visual information via optical character recognition in a timely manner was useful to ensure that ED staffs are fully prepared to receive patients. For ED staff, timely monitoring and understanding of the situation offered by the EMS were useful for maintaining a high quality of clinical practice.

### Comparison With Prior Work

Appropriate use of medical apps could lead to a seamless transition of management from prehospital to post-ED arrival. As reported in this study, apps can be used to obtain information such as medical history and prehospital electrocardiograms in advance. From such information, physicians can prepare for urgent interventions (eg, catheterization) before the patient arrives at the hospital. There have been several reports on the usefulness of apps that provide prehospital information in emergency medicine. A system called ORION (Osaka Emergency Information Research Intelligent Operation Network system), introduced in Osaka City in 2013, reduced the number of cases that are difficult to transport [12]. Another study reported that the communication-type medical apps can be accurately used remotely, and information can be shared with the stroke team to prepare for rapid treatment [5,20,21].

The NSER mobile app is a digitalization tool for EMS in the clinical setting, and there are no patients for whom the app cannot be used. Nevertheless, we think that the system is more suitable for patients who need emergency interventions (eg, cardiac catheterization) [2-5,7] rather than those with cardiac arrest (EMS may not have enough time to use the app). Due to the limited sample size, we could not analyze data after stratifying by these variables. Thus, we are conducting further study in different settings to examine the effectiveness of the app.

### Strengths and Limitations

This study has several strengths, a few of which are as follows. First, there were no similar studies on prehospital information transfer apps aimed at improving the efficiency of emergency patient transport without assuming a specific disease. Second, the interrupted time-series analysis estimates the effect of intervention on a population and is a study design without a control group. Third, the advantage of reducing phone-communication time through real-time information sharing is noticeable especially when the EMS is consulting multiple hospitals to accept patients at the same time, not to mention that in many cases it is difficult to transport patients, particularly in urban areas in Japan. Indeed, according to statistics from the Ministry of Health, Labor, and Welfare in 2016, even for critically ill patients, there were 10,039 cases (2.3%) in which the number of consultations to medical institutions was ≥4 times and 22,104 cases (5.0%) in which the time spent on site was 30 minutes or longer [22]. In such cases, given the tough negotiation with hospitals, the reduction of phone-communication time while efficiently sharing prehospital information should reduce the burden on EMS. We believe that the findings from this study allow us to consider the substantial contribution and potential benefits of mobile apps to emergency medical care.

Our study has several limitations. First, although we performed a 3-hour lecture for using the app prior to implementation, users may not have been able to get accustomed to the app quickly enough in the clinical setting. Despite this, there was clear improvement in phone-communication time immediately after its implementation. Second, there was no control group in our ITSA design [23]. Nonetheless, our findings are likely robust, given that there were no interventions other than the implementation of the app that may have affected the outcome. While the COVID-19 pandemic may have affected the assumptions of the interrupted time series analysis, the implementation of personal protective equipment for EMS was initiated on March 2, 2020. Therefore, change in practice due to the pandemic may not have substantially affected the EMS during the study period (July 8, 2021, to October 27, 2021). Third, we did not have information on the time to intervention at the ED (eg, time to urgent catheterization) and ED overcrowding. Therefore, further studies are needed to examine the impact of the app on clinical practice. Fourth, in this study, we only evaluated the observed values for 8 weeks before and after the intervention. A recent simulation-based study on ITSA reported that 12 preintervention and 12 postintervention time points may be required for a moderate intervention effect sizes [24]. Lastly, there is limited generalizability of our findings because our study was a single-center, retrospective observational study in Japan with a small sample size. In addition, EMS systems are different across countries. The extrapolation of our findings to other settings should be done with caution, and therefore additional large-scale studies are warranted.

### Future Directions

As shown in the 2 cases, the implementation of a mobile app for efficient real-time sharing of prehospital patient information has potential to reduce the time to intervention, resulting in better patient outcomes. In addition, in Japan, especially in the urban areas, there is the difficulty in determining the hospital for emergency patient [1]; however, for instance, the average number of hospitals that EMS phoned to transport patient was 1 in Kamakura city during this study period, so the decision to transport a patient did not take extra time. Therefore, the app may reduce the overall transportation time by decreasing the number of calling from EMS to hospitals.

### Conclusions

The implementation of a mobile app for EMS reduced phone-communication time by 22% without increasing mortality or overall transportation time despite the implementation of new methods in the real clinical setting. Real-time patient information sharing, such as the transfer of monitor images and photos of the accident site, could facilitate optimal patient care and resource use.

## Appendix

Multimedia Appendix 1 Workflow of an emergency medical services team using NEXT Stage ER mobile.

Multimedia Appendix 2 An example of sharing trauma images, monitors, and clinical information from emergency medical services to the emergency department staff.

Multimedia Appendix 3 Monitor screen image of a case of a 47-year-old man complaining of chest pain.

Multimedia Appendix 4 The damaged bicycle image of a case of a 35-year-old man with trauma.

Multimedia Appendix 5 Left lower extremity injuries of a case of a 35-year-old man with trauma.

Multimedia Appendix 6 Monitor screen image of a case of a 35-year-old man with trauma.

## Abbreviations

ED: emergency department
EMS: emergency medical services
ITSA: Interrupted time-series analysis
NSER mobile: NEXT Stage ER mobile
