# Supplementary figures and images for: Experimental Implementation of NSER Mobile App for Efficient Real-Time Sharing of Prehospital Patient Information With Emergency Departments: Interrupted Time-Series Analysis
Source: JMIR Form Res. 2022 Jul 6;6(7):e37301. doi: 10.2196/37301 (PMC9301553; doi:10.2196/37301)

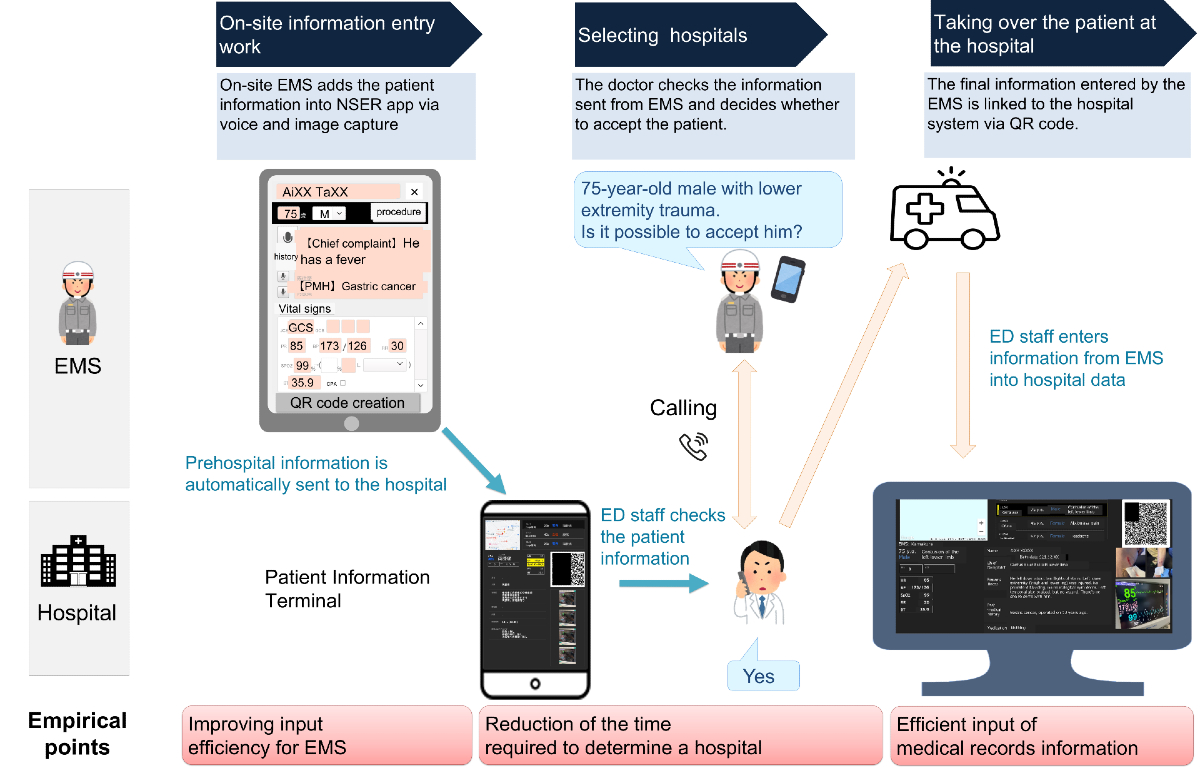

Supplement: Multimedia Appendix 1 [file formative_v6i7e37301_app1.png]

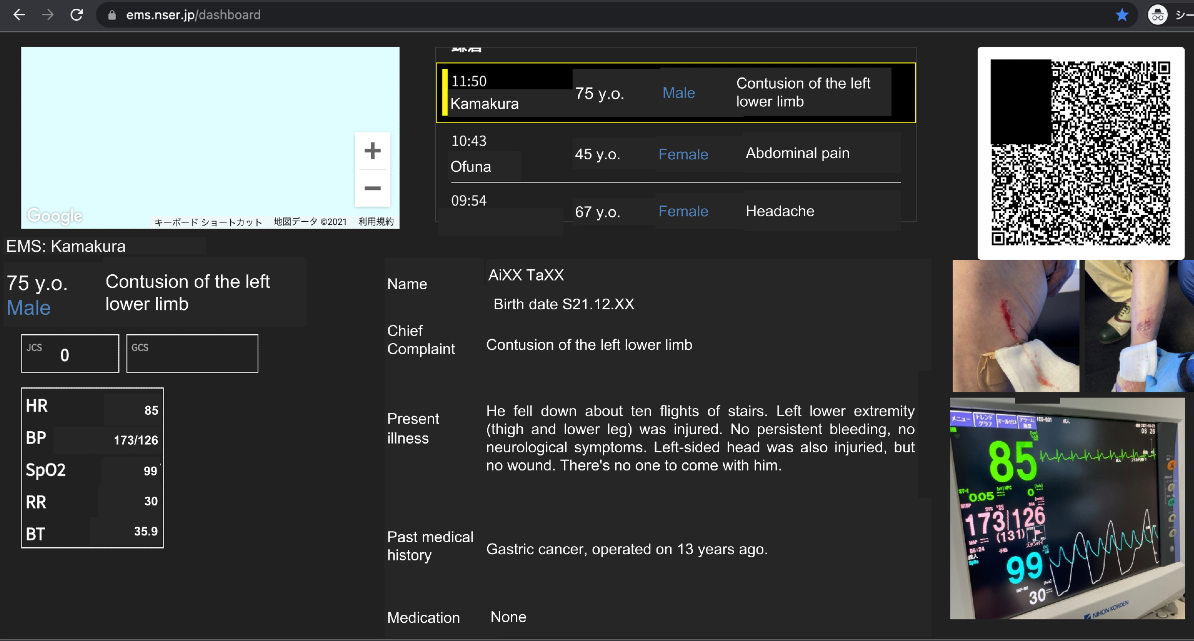

Supplement: Multimedia Appendix 2 [file formative_v6i7e37301_app2.png]

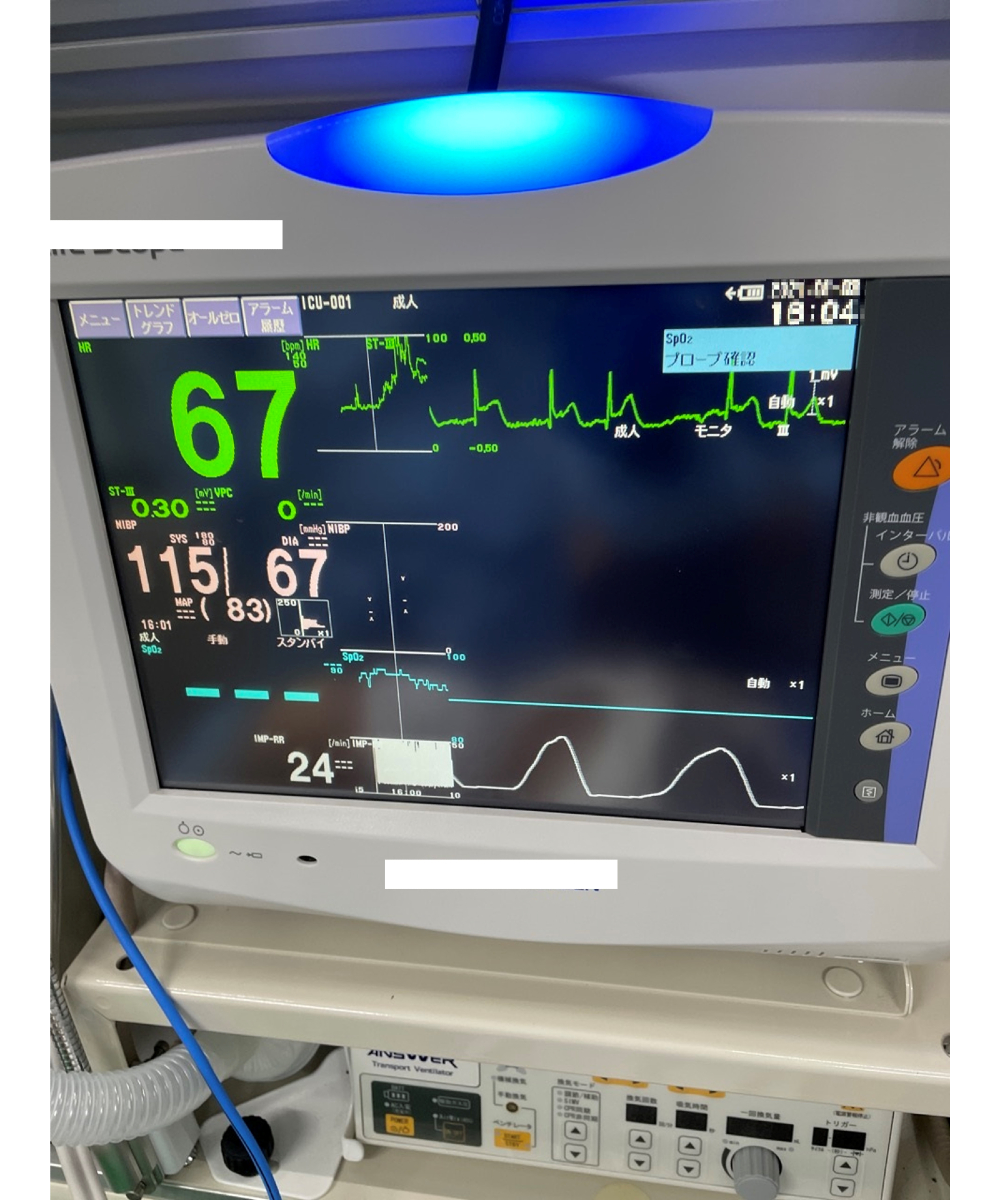

Supplement: Multimedia Appendix 3 [file formative_v6i7e37301_app3.png]

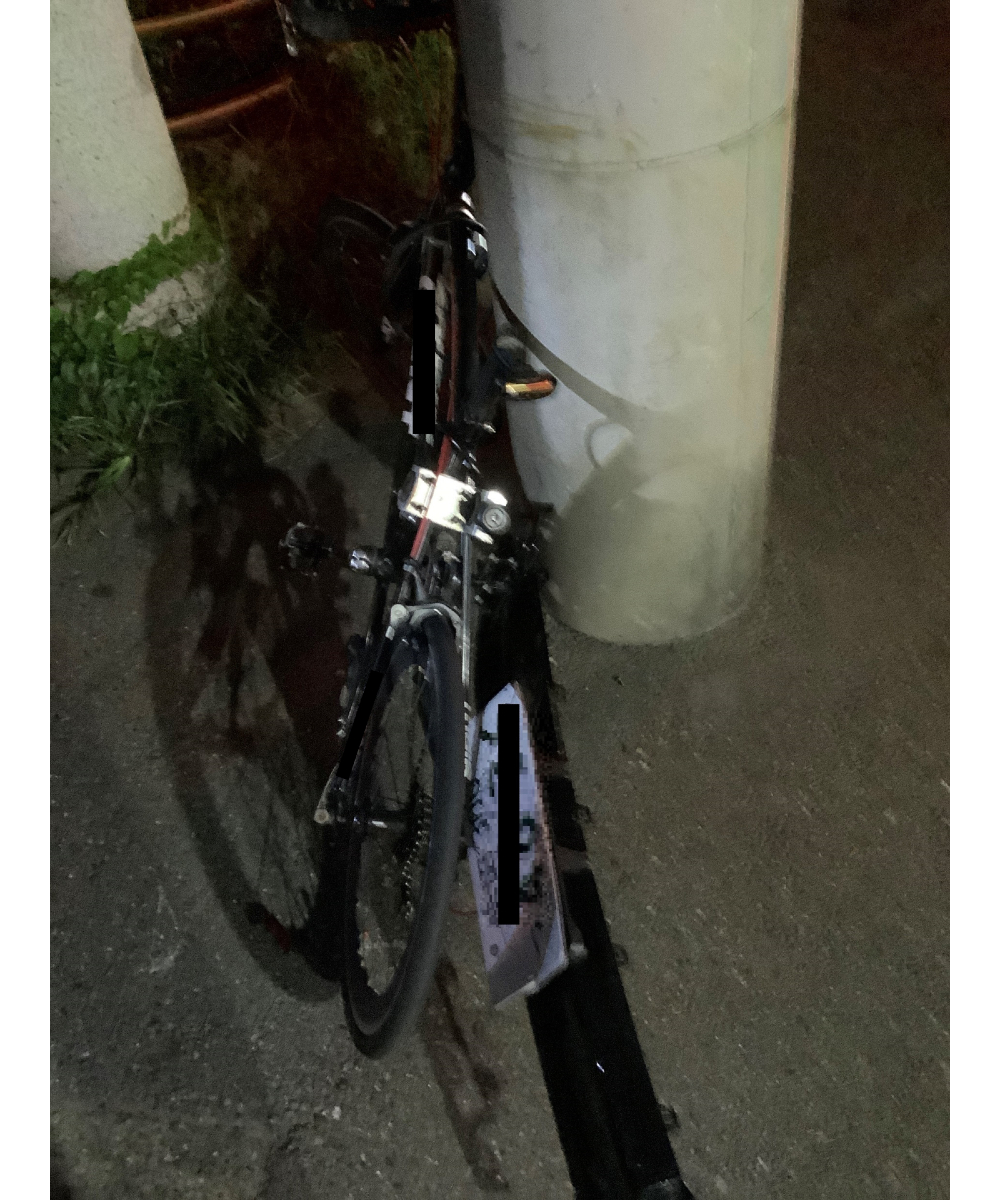

Supplement: Multimedia Appendix 4 [file formative_v6i7e37301_app4.png]

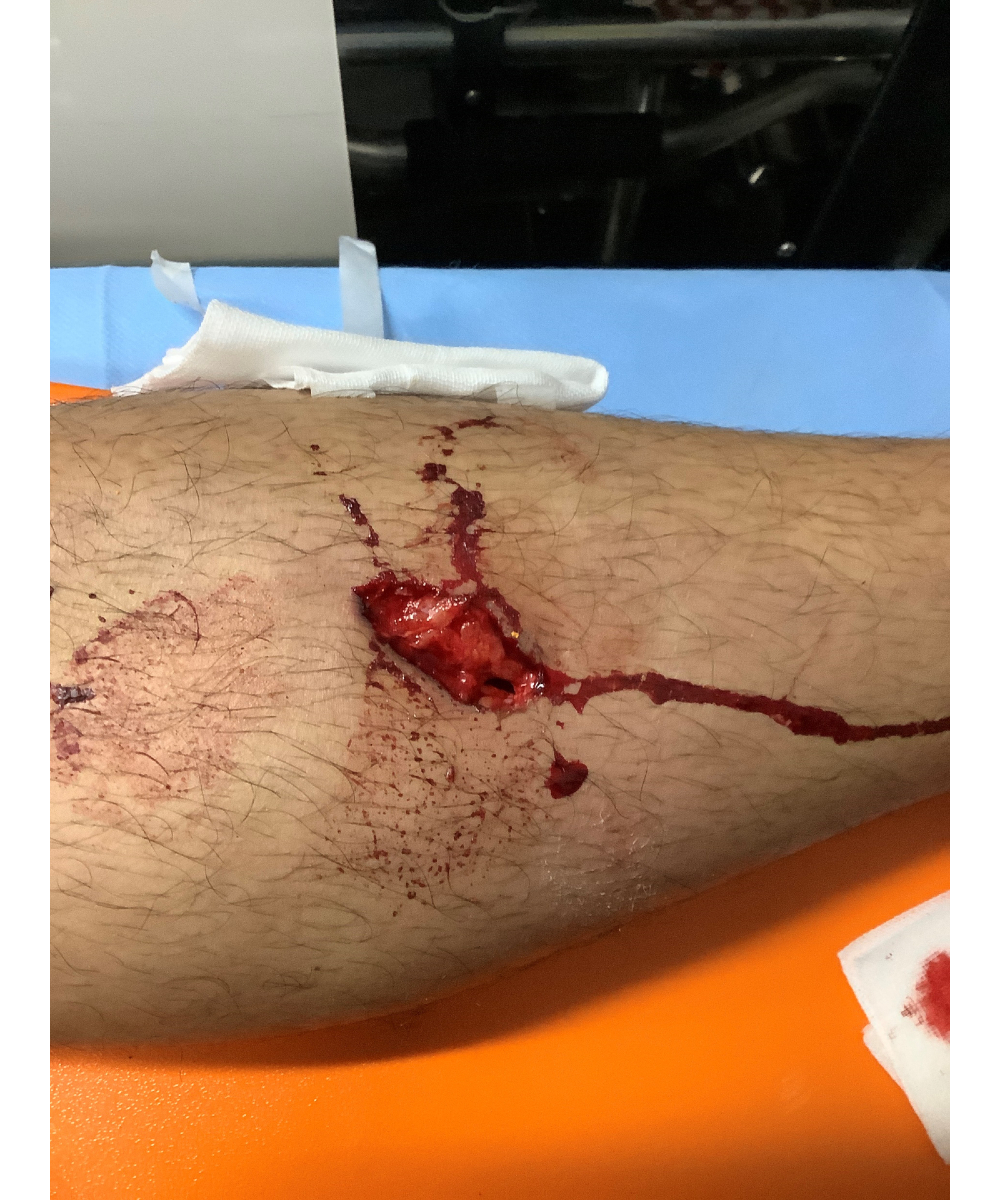

Supplement: Multimedia Appendix 5 [file formative_v6i7e37301_app5.png]

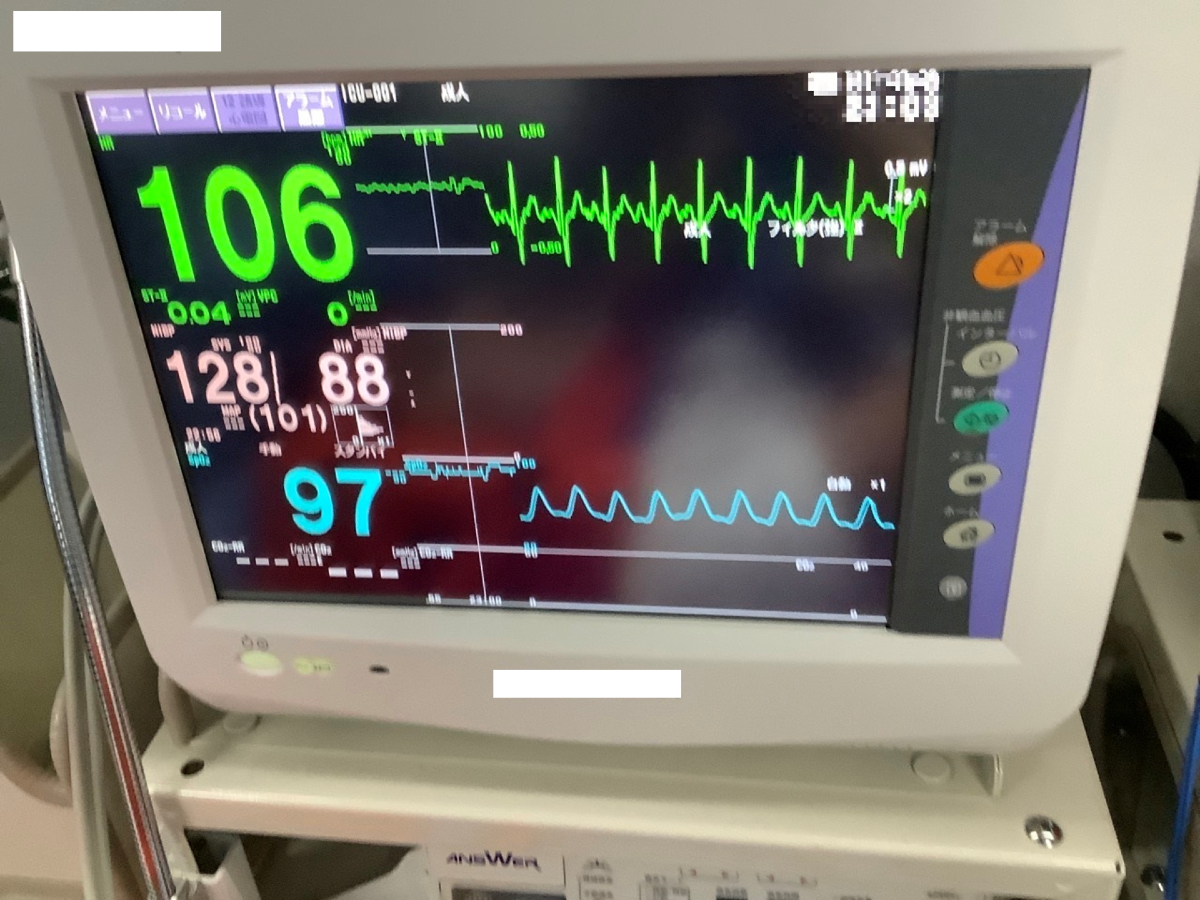

Supplement: Multimedia Appendix 6 [file formative_v6i7e37301_app6.png]
